# Perisphaerinae Brunner von Wattenwyl and *Hyposphaeria* Lucas are valid names concealed by the unavailable names Perisphaeriinae and *Perisphaeria* Burmeister (Blattodea, Blaberidae)

**DOI:** 10.3897/zookeys.574.7063

**Published:** 2016-03-28

**Authors:** Xin-Ran Li, Zong-Qing Wang

**Affiliations:** 1 Institute of Entomology, College of Plant Protection, Southwest University, Beibei, Chongqing 400716, China

**Keywords:** cockroach, nomenclature, spelling, *Perisphaerus*, *Perisphaera*, Perisphaeriidae, Perisphaeridae

## Abstract

The incorrect generic name *Perisphaeria* Burmeister, 1838 results from a mistake by Princis (1947). *Perisphaeria* is an incorrect spelling by Burmeister referring to *Perisphaerus* Serville, 1831. Princis (1947) incorrectly regarded *Perisphaeria* as proposed by Burmeister and as the senior synonym of *Hyposphaeria* Lucas, 1863, which is valid. The subfamilial name Perisphaeriinae based on *Perisphaeria* should be corrected to Perisphaerinae Brunner von Wattenwyl, 1865 based on *Perisphaerus*; but note Princis’ Perisphaeriinae is based on his *Perisphaeria* Burmeister. The taxa involved are listed.

## Introduction

Perisphaeriinae and its type genus *Perisphaeria* are unavailable. *Perisphaeria* was never proposed as a new genus, but is an incorrect spelling of *Perisphaerus* Serville, 1831 by Burmeister (1838: 483). It is thus unavailable (ICZN 1999: Article 33.3), although it may be a Latinized spelling of *Périsphère* Serville, 1831, i.e. an unjustified emendation of *Perisphaerus*, by Burmeister (ICZN 1999: Article 33.2 and 33.5); the family name is therefore unavailable and should be corrected (ICZN 1999: Article 35.4.1). The genus *Perisphaeria* revised by Princis (1947: 51) refers to *Hyposphaeria* Lucas, 1863, which is valid but Princis regarded it as a junior synonym of *Perisphaeria* Burmeister of his own concept.

*Perisphaeria* was attributed to Burmeister by Princis (1947: 51). Here arises the question: did Burmeister establish such a genus or is it simply an unjustified emendation? Burmeister (1838: 483) added an asterisk to *Perisphaeria* with which he usually indicated a new taxon. However, it was normal practice for Burmeister to then provide an etymology for the proposed new name, but this was not the case for *Perisphaeria*, suggesting that this genus was not described as new. Secondly, Burmeister (1838: 484) included four subgenera in *Perisphaeria*, of which the first one (nominotypical subgenus) is *Perisphaeria* Serv., and doubted their ability to roll into a ball as described by Serville, whilst Serville (1831: 44) used this as a diagnostic character and as the etymology for his *Perisphaerus*. Thus Burmeister must have his *Perisphaeria* attributed to Serville was in fact *Perisphaerus*. Thirdly, *Perisphaeria* is not an emendation of *Perisphaerus* according to the rules of the Code (ICZN 1999: Article 33.2.1), i.e., there is no explicit statement of intention, *Perisphaerus* was not cited and replaced, and no similar case of treatment is found in the same publication. Therefore, *Perisphaeria* is deemed to be an incorrect subsequent spelling and thus unavailable (ICZN 1999: Article 33.3). It can be speculated that the asterisk annotation to *Perisphaeria* (Burmeister 1838: 483) was not only used by Burmeister to denote a new name, but also to denote names with new scope or definition, i.e. extending the scope of *Perisphaerus* to include species from both Asia and Africa. The species Burmeister (1838) described were from Africa and not Asia, from which Serville (1831: 44) described *Perisphaerus armadillo*; that is why he did not accept Serville’s words. It is not impossible, however, that this asterisk is simply a mistake, especially when such a mark is absent next to the *Perisphaeria* in the key (Burmeister 1838: 481).

We speculate that *Perisphaeria* may be, in a practical but not a nomenclatural sense, a Latinized version of *Périsphère* Serville, 1831. In other words, an emendation of *Perisphaerus* rather than an incorrect spelling of *Perisphaerus*, because French names seemed to be more formal at that time. However, Serville (1831: 44) himself gave a Latinized (i.e. valid) version which was exactly *Perisphaerus*, just following PÉRISPHÈRE and followed by the Greek etymology; but such a change in spelling by Burmeister is not demonstrably intentional, thus not an emendation in nomenclatural sense (ICZN 1999: Article 33.2.1). Similarly, *Perisphaera* is also a Latinized version of *Périsphère*, being used by Serville (1839: 132) himself for the first time. The word ball is a feminine noun in both French (sphère) and Latin (*sphaera*); therefore *Perisphaerus* is a result of incorrect Latinization by Serville (1831) when he Latinized *Périsphère* the moment he proposed it. Subsequently he realized that and re-Latinized the French name in a later paper (Serville 1839). However, *Perisphaerus* should remain in use rather than *Perisphaera* since incorrect Latinization is not to be corrected (ICZN 1999: Article 32.5.1).

No matter which name, or to be more accurate, which spelling—*Perisphaerus*, *Perisphaera* or *Perisphaeria*—the family name is based on, the name established by Brunner von Wattenwyl (1865: 303) should be corrected to Perisphaeridae (ICZN 1999: Article 32.5.3.3). Note also that Brunner v. W. (1865) proposed it at the rank of tribe, which should be corrected to Perisphaerini under current nomenclature. The situation is further complicated by Princis (1947: 51) who attributed *Perisphaeria* to Burmeister and regarded it as restricted to African species, whilst the Asian species were re-combined with *Perisphaerus* Serville. Princis (1960: 438) subsequently included *Perisphaeria* Burmeister in Perisphaeriidae and established a separate family, Derocalymmidae, to include *Perisphaerus*. As a result, the true type genus of Perisphaeriidae was transferred to another family, but one of its incorrect spellings and wrong authorship remained; Perisphaeriidae is thus no longer correct. Perisphaeriidae and Derocalymmidae are now considered synonyms (Roth 2003), whilst Princis’ erroneous use of the genus-group names has become widespread since his catalogue (Princis 1964). It is noteworthy that Princis’ Perisphaeriidae has the same spelling as Perisphaeriidae Brunner v. W. that was based on the incorrect spelling *Perisphaeria* Serville. These two names, identical in spelling and similar in scope, are inherently different.

Before Princis (1947), most authors were well aware of the concept of these generic names (spellings), and cited correct authorship (i.e. Serville) of *Perisphaerus* when referring to it under its incorrect spelling (*Perisphaeria*), though an exception is Fischer (1853: 94) who attributed *Perisphaeria* to Burmeister. However, he gave *Perisphaera* Serv. as a synonym, which suggests that Fischer understood well the concept of them but cited an incorrect spelling with its author instead of the original spelling with its author. Lucas (1863: 408) recognized Burmeister’s *Perisphaeria* as the same name as *Perisphaera* Serville, but suggested that they cannot refer to each other, i.e., he disputed the extension of its scope. Lucas stated that *Perisphaera* should be restricted to the Asian species which can roll into a ball. Lucas proposed a new genus *Hyposphaeria* Lucas, 1863 to include African species that do not roll into a ball and included species described by Burmeister. Brunner v. W. (1865), Walker (1868), Saussure and Zehntner (1895) and Kirby (1904) all treated *Perisphaeria* Serville as valid and provided synonyms including *Perisphaera* and/or *Perisphaerus*; it is inferred from this that they accepted Burmeister’s spelling to be the valid name of the genus proposed by Serville, and that *Perisphaeria* and Perisphaeriidae were once in prevailing usage. Brunner v. W. (1865) and Walker (1868) did not mention *Hyposphaeria*, while Saussure and Zehntner (1895) agreed with Lucas and included only Asian ‘rollable’ species in *Perisphaeria* Serville. However, they did not adopt *Hyposphaeria* since they believed species in this genus should be placed in various genera. Kirby (1904) in his catalogue treated *Hyposphaeria* as a valid name and included four species in it, with *Hyposphaeria stylifera* (Burmeister, 1838) designated as type species.

In summary, *Perisphaerus* Serville, 1831 is a valid name and includes the ‘rollable’ species, mostly from the Oriental Region, with *Perisphaera* and *Perisphaeria* as unavailable spellings, although the latter spelling was once enlarged in its scope and widely used. *Hyposphaeria* Lucas, 1863 is a valid name and includes the African ‘unrollable’ species, of which the early ones were described under *Perisphaeria* by Burmeister (1838). This name was once incorrectly synonymized with the unavailable name *Perisphaeria* Burmeister by Princis (1947). Perisphaeridae Brunner v. W., 1865 is a valid family name based on *Perisphaerus* Serville. Perisphaeriidae Brunner v. W. is unavailable since it was based on *Perisphaeria* Serville. The Perisphaeriidae used by Princis has the same spelling but is not identical since this name was based on *Perisphaeria* Burmeister which is a consequence of his misunderstanding.

Princis’ erroneous revision of the names mentioned above should be no longer adopted.

Serville signed his name as J. G. Audinet-Serville in his published works and some authors attributed the scientific names established by him to Audinet-Serville; however, cockroach taxonomists (e.g. those mentioned above) prefer Serville in citation and authorship of names, and as such this convention is followed to avoid ambiguity. The abbreviation Brunner v. W. for Brunner von Wattenwyl is used for brevity.

## Appendix

Synonymy and taxonomic inclusion of names mentioned

**Family Blaberidae**

**Subfamily Perisphaerinae Brunner von Wattenwyl, 1865**

Perisphaeridae Brunner von Wattenwyl, 1865: 302, as a tribe, type genus *Perisphaeria* Serv..

Perisphaeridae Walker, 1868: 168, type genus *Perisphaeria*. [not Perisphaeridae Brunner von Wattenwyl]

Périsphaeriens: Saussure and Zehntner 1895: 1, as a tribe.

Perisphaeriinae: Kirby 1904: 179.

Derocalymmidae Princis, 1960: 438; Roth 2003: 102, as a synonym of Perisphaeriinae.

Derocalymminae Princis, 1960: 438; Roth 2003: 102, as a synonym of Perisphaeriinae.

Perisphaeriidae Princis, 1960: 438, type genus *Perisphaeria* Burmeister.

Perisphaeriinae Princis, 1960: 438, type genus *Perisphaeria* Burmeister; Roth 2003: 41.

Genera included (Roth 2003): *Bantua*, *Compsagis*, *Cyrtotria*, *Derocalymma*, *Ellipsica*, *Elliptoblatta*, *Gymnonyx*, *Hostilia*, *Hyposphaeria* (*sic*: *Perisphaeria* Burmeister), *Laxta*, *Neolaxta*, *Perisphaerus*, *Pilema*, *Platysilpha*, *Poeciloblatta*, *Pseudoglomeris*, *Pseudocalolampra*, *Trichoblatta*, *Zuluia*.

**Genus *Perisphaerus* Serville, 1831**

*Perisphaerus* Serville, 1831: 44, type species *Perisphaerus armadillo* Serville, 1831, by monotypy; Princis 1947: 51.

*Perisphaeria*: Burmeister 1838: 483; Lucas 1863: 408; Brunner von Wattenwyl 1865: 303; Walker 1868: 168; Saussure and Zehntner 1895: 12, 32; Kirby 1904: 189.

*Perisphaera*: Serville 1839: 132; Fischer 1853: 94, as a synonym of *Perisphaeria* Burmeister; Lucas 1863: 408; Brunner von Wattenwyl 1865: 303; Walker 1868: 168.

*Perisphaeria* Burmeister: Fischer 1853: 94.

Species included (Princis 1964; 1971; Roth 2003): *aeneus*, *altus*, *armadillo*, *aterrimus*, *brunneri*, *contiguus*, *cotesianus*, *flavicornis*, *flavipes*, *flexicollis*, *glomeriformis*, *inaequalis*, *multipunctatus*, *pubescens*, *punctatus*, *rubescens*, *semilunatus*.

**Genus *Hyposphaeria* Lucas, 1863**

*Hyposphaeria* Lucas, 1863: 408; Kirby 1904: 193, *Hyposphaeria stylifera* (Burmeister, 1838) was selected as type species.

*Perisphaeria* Burmeister: Princis 1947: 51.

Species included (Princis 1964; 1971; Roth 2003): *aspera*, *basuto*, *bicolor*, *carlgreni*, *guillarmodi*, *hancocki*, *hirta*, *hirtula*, *micans*, *mucronata*, *nodosa*, *peringueyi*, *pilifera*, *pilosa*, *rudebecki*, *ruficornis*, *saussurei*, *saxicola*, *scabra*, *scabrella*, *stylifera*, *substylifera*, *verrucosa*, *virescens*.
